# The Generalized Euler Characteristics of the Graphs Split at Vertices

**DOI:** 10.3390/e24030387

**Published:** 2022-03-09

**Authors:** Omer Farooq, Michał Ławniczak, Afshin Akhshani, Szymon Bauch, Leszek Sirko

**Affiliations:** Institute of Physics, Polish Academy of Sciences, Aleja Lotników 32/46, 02-668 Warsaw, Poland; akhshani@ifpan.edu.pl (A.A.); bauch@ifpan.edu.pl (S.B.)

**Keywords:** quantum graphs, microwave networks, Euler characteristic, Neumann and Dirichlet boundary conditions

## Abstract

We show that there is a relationship between the generalized Euler characteristic $E_{o}\left(\right|V_{D_{o}}\left|\right)$ of the original graph that was split at vertices into two disconnected subgraphs i=1,2 and their generalized Euler characteristics $E_{i}\left(\right|V_{D_{i}}\left|\right)$. Here, $|V_{D_{o}}|$ and $|V_{D_{i}}|$ denote the numbers of vertices with the Dirichlet boundary conditions in the graphs. The theoretical results are experimentally verified using microwave networks that simulate quantum graphs. We demonstrate that the evaluation of the generalized Euler characteristics $E_{o}\left(\right|V_{D_{o}}\left|\right)$ and $E_{i}\left(\right|V_{D_{i}}\left|\right)$ allow us to determine the number of vertices where the two subgraphs were initially connected.

## 1. Introduction

The concept of graphs was already introduced in the XVIII century by Leonhard Euler [1]. Two hundred years later, Linus Pauling [2] considered quantum graphs in order to describe the motion of quantum particles in a physical network. The models of quantum graphs were widely used to investigate many physical systems, e.g., quantum wires [3], mesoscopic quantum systems [4,5], a topological edge invariant [6], and the photon number statistics of coherent light [7]. Broad applications of graphs and networks mean that the theory of quantum graphs has been a subject of extensive research [8,9,10,11,12,13,14].

We will consider a metric graph Γ=(V,E), which consists of *v* vertices, v∈V, connected by *e* edges, e∈E. The edges *e* are intervals of the length $l_{e}$ on the real line R. The metric graph becomes quantum when we equip it with the free Schrödinger operator. In our case, this is the one-dimensional Laplace operator, which equals $L\left(\Gamma \right)=-\frac{d^{2}}{dx_{e}^{2}}$ on each of the edges e∈E of the graph Γ. The self-adjoint Laplace operator L(Γ) has a discrete and non-negative spectrum [12].

A signal inside a graph moves along the edges, and at each vertex v∈V it splits and enters all edges adjacent to *v*. If the signal enters the vertex *v* along the edge $e^{\prime }$ and leaves it along the edge *e*, then the ratio of amplitudes of entering and leaving signals is given by the vertex scattering matrix, which depends on the vertex boundary condition. We will consider two types of vertex boundary conditions. The standard boundary conditions are called also Neumann boundary conditions, for which the eigenfunctions are continuous at vertices and the sums of their oriented derivatives at vertices are zero. The vertex scattering matrix corresponding to the Neumann boundary conditions [15] is given by

(1) $${}^{N}\sigma_{e,e^{\prime }}^{\left(v\right)}=\frac{2}{d_{v}}-\delta_{e,e^{\prime }},$$

where $d_{v}$ is the degree of the vertex *v*, i.e., the number of edges incident to the vertex *v*, and $\delta_{e,e^{\prime }}$ is the Kronecker delta. The vertices with the Neumann boundary conditions will be denoted as $v_{N}$.

For the Dirichlet boundary condition, an eigenfunction at the vertex takes the value zero, which leads to the vertex scattering matrix [15,16]

(2) $$\begin{array}{c} {}^{D}\sigma_{e,e^{\prime }}^{\left(v\right)}=-\delta_{e,e^{\prime }}. \end{array}$$

One should point out that the Dirichlet boundary conditions are imposed only at degree one vertices and higher-degree Dirichlet vertices should be treated as separate degree one Dirichlet vertices. The vertices with the Dirichlet boundary conditions will be denoted as $v_{D}$. Different types of the boundary conditions, including the Neumann and Dirichlet ones for higher-dimensional systems such as grains, are comprehensively described in Refs. [17,18].

The total number of vertices |V| in a general graph, consisting of both Neumann and Dirichlet boundary conditions, is defined by $\left|V|=\right|V_{N}\left|+\right|V_{D}|$, where $|V_{N}|$ and $|V_{D}|$ denote the number of vertices with Neumann and Dirichlet boundary conditions, respectively.

One of the most important characteristics of metric graphs Γ=(V,E) with the standard boundary conditions ($|V_{D}|=0$) is the Euler characteristic

(3) χ=|V|−|E|,

where |V| and |E| denote the number of vertices and edges of the graph. It is a purely topological quantity; however, it has been shown in [19,20,21,22] that it can also be defined by the graph and microwave network spectra. The formula describing the generalized Euler characteristic E [22,23], which is also applicable for graphs and networks with the Dirichlet boundary conditions, will be discussed later.

In the experimental investigation of properties of quantum graphs, we used microwave networks simulating quantum graphs [16,24,25,26,27,28,29]. The emulation of quantum graphs by microwave networks is possible because of the formal analogy of the one-dimensional Schrödinger equation describing quantum graphs and the telegrapher’s equation for microwave networks [24,26]. Microwave networks are the only ones that allow for the experimental simulation of quantum systems with all three types of symmetry within the framework of the random matrix theory (RMT): Gaussian orthogonal ensemble (GOE)—systems with preserved time reversal symmetry (TRS) [16,21,24,25,27,30,31,32], Gaussian unitary ensemble (GUE)—systems with broken TRS [24,28,33,34,35,36], and Gaussian symplectic ensemble (GSE)—systems with TRS and half-spin [37]. The other model systems, which are not as versatile as microwave networks, but are often used in simulations of complex quantum systems, are flat microwave billiards [38,39,40,41,42,43,44,45,46,47,48,49,50,51,52,53,54], and exited atoms in strong microwave fields [55,56,57,58,59,60,61,62,63,64,65,66,67].

In this article, we will analyze the splitting of a quantum graph (network) into two disconnected subgraphs (subnetworks). Using a currently introduced spectral invariant—the generalized Euler characteristic E[22]—we determine the number $|V_{c}|$ of common vertices where the two subgraphs were initially connected. The application of the generalized Euler characteristic E for this purpose stems from the fact that it can be evaluated without knowing the topologies of quantum graphs (networks), using small or moderate numbers of their lowest eigenenergies (resonances). The theoretical results are numerically verified and confirmed experimentally using the spectra of microwave networks simulating quantum graphs.

## 2. Theoretical Outline

### 2.1. The Generalized EULER Characteristic

In Refs. [21,22], the formulas for the Euler characteristic for graphs with the standard boundary conditions at the vertices and with the mixed ones, standard and Dirichlet boundary conditions at vertices, were derived. In the case of the standard boundary conditions,

(4) $$\chi =2+8\pi^{2}\sum_{\begin{array}{c} k_{n}\in \Sigma \left(L^{\mathrm{st}}\left(\Gamma \right)\right)\\ k_{n}\neq 0 \end{array}}\frac{\sin(k_{n}/t)}{\left(k_{n}/t\right)\left({\left(2\pi \right)}^{2}-{\left(k_{n}/t\right)}^{2}\right)}{|}_{t\geq t_{0}},$$

where $\Sigma (L^{\mathrm{st}}\left(\Gamma \right))$ denotes the spectrum of the Laplacian $L^{\mathrm{st}}\left(\Gamma \right)$ with the standard vertex conditions, taken in the square root scale, i.e., the numbers $k_{n}$ are the square roots of the eigenenergies $\lambda_{n}$ and *t* is a scaling parameter [19,20,21] with $t_{0}=\frac{1}{2l_{min}}$, where $l_{min}$ is the length of the shortest edge of the graph. The above formula is equivalent to Equation (3); however, instead of using topological information about graphs or networks, such as the number of vertices |V| and edges |E|, it requires a certain number of the lowest eigenenergies (resonances) of graphs or networks.

For graphs and networks with the mixed boundary conditions, namely the standard and Dirichlet ones ($|V_{D}|\neq 0$), the generalized Euler characteristic can be expressed by the following formula:

(5) $$\chi_{G}:=\chi -|V_{D}{\left|=8\pi^{2}\sum_{k_{n}\in \Sigma \left(L^{\mathrm{st},D}\left(\Gamma \right)\right)}\frac{\sin(k_{n}/t)}{\left(k_{n}/t\right)\left({\left(2\pi \right)}^{2}-{\left(k_{n}/t\right)}^{2}\right)}\right|}_{t\geq t_{0}}.$$

In Equation (5), the spectrum of the Laplacian $L^{\mathrm{st},D}\left(\Gamma \right)$ with the standard and Dirichlet vertex conditions is denoted by $\Sigma (L^{\mathrm{st},D}\left(\Gamma \right))$.

The above two equations can be unified into a single one for the generalized Euler characteristic:

(6) $$E(|V_{D}\left|\right)=2\delta_{0,|V_{D}|}+8\pi^{2}\sum_{\begin{array}{c} k_{n}\in \Sigma \left(L\left(\Gamma \right)\right)\\ k_{n}\neq 0 \end{array}}\frac{\sin(k_{n}/t)}{\left(k_{n}/t\right)\left({\left(2\pi \right)}^{2}-{\left(k_{n}/t\right)}^{2}\right)}{|}_{t\geq t_{0}}.$$

Depending on the boundary conditions, Σ(L(Γ)) denotes either the spectrum of the Laplacian $L^{\mathrm{st}}\left(\Gamma \right)$ or $L^{\mathrm{st},D}\left(\Gamma \right)$. In the borderline cases $|V_{D}|=0$ and $|V_{D}|\neq 0$, $E(|V_{D}|=0)=\chi$ and $E(|V_{D}\left|\neq 0\right)=\chi_{G}$, recovering, respectively, Equations (4) and (5).

From the experimental point of view, the usefulness of Equation (6) stems from the fact that the generalized Euler characteristic can be evaluated using only a limited number $K=K_{min}$ of the lowest eigenvalues (resonances) [21,22,68,69]

(7) $$K\geq \left|V\right|+2Lt{\left[1-\exp\left(\frac{-\epsilon \pi }{Lt}\right)\right]}^{-1/2},$$

where |V| is the total number of graph vertices, $L=\sum_{e\in E}l_{e}$ is the total length of the graph, and ϵ is the accuracy of determining the Euler characteristic from Formula (7). To obtain the smallest possible number of resonances $K_{min}$, for a given accuracy ϵ, we assign to *t* its smallest allowed value $t=t_{0}=\frac{1}{2l_{min}}$. Since the Euler characteristic is an integer, the accuracy of its determination should be taken ϵ<1/2. In our calculations of $K_{min}$, we assumed ϵ=1/4.

### 2.2. A Graph Split into Two Disconnected Subgraphs

In order to simplify the description of the graphs, we introduce the following notation of graphs and networks $\Gamma (|V|,|E|,|V_{D}|)$, where $\left|V|=\right|V_{N}\left|+\right|V_{D}|$. A graph or network $\Gamma (|V|,|E|,|V_{D}|)$ contains |V| vertices, including $|V_{N}|$ and $|V_{D}|$ vertices with standard (Neumann) and Dirichlet boundary conditions and |E| edges.

**Figure 1:**
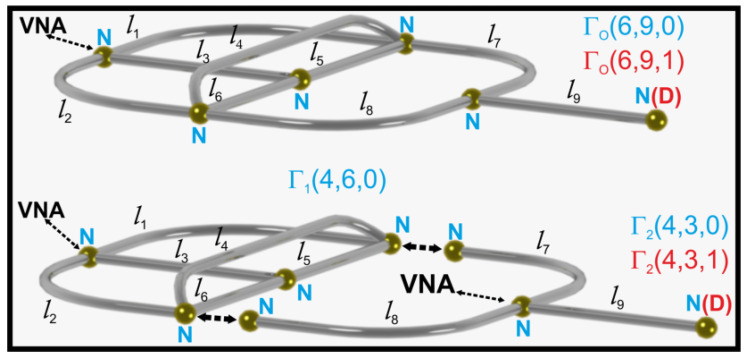
The scheme of the original graph $\Gamma_{o}\left(6,9,0\right)$, which was divided into two subgraphs $\Gamma_{1}\left(4,6,0\right)$ and $\Gamma_{2}\left(4,3,0\right)$. All graphs possess the vertices with the Neumann boundary conditions, which are marked by blue capital letters *N*. In the case of the graphs with the mixed boundary conditions, the original graph $\Gamma_{o}\left(6,9,1\right)$ was divided into two subgraphs $\Gamma_{1}\left(4,6,0\right)$ and $\Gamma_{2}\left(4,3,1\right)$. The vertices with the Dirichlet boundary conditions are marked by red capital letters *D*. The vertices where a vector network analyzer was connected to the microwave networks simulating quantum graphs presented in this figure are marked by VNA.

We will consider a general situation when an original graph $\Gamma_{o}\left(\right|V_{o}\left|,\right|E_{o}\left|,\right|V_{D_{o}}\left|\right)$ is split into two disconnected subgraphs $\Gamma_{i}\left(\right|V_{i}\left|,\right|E_{i}\left|,\right|V_{D_{i}}\left|\right)$, i=1,2, at the common for the subgraphs vertices $V_{c}$, which are characterized by the Neumann boundary conditions. In the partition process, each common vertex $v\in V_{c}$ will be split into two new vertices belonging to the different subgraphs (see Figure 1).

The generalized Euler characteristics of the original graph and its subgraphs are $E_{o}\left(\right|V_{D_{o}}\left|)=\right|V_{o}\left|-\right|E_{o}\left|-\right|V_{D_{o}}|$ and $E_{i}\left(\right|V_{D_{i}}\left|)=\right|V_{i}\left|-\right|E_{i}\left|-\right|V_{D_{i}}|$, i=1,2, respectively. The relationships between the number of vertices and edges of the graphs are the following: $|V_{o}\left|+\right|V_{c}\left|=\right|V_{1}\left|+\right|V_{2}|$, $|E_{o}\left|=\right|E_{1}\left|+\right|E_{2}|$. It leads to the following relationship between $E_{o}\left(\right|V_{D_{o}}\left|\right)$ and $E_{i}\left(\right|V_{D_{i}}\left|\right)$, i=1,2

(8) $$E_{1}\left(\right|V_{D_{1}}\left|\right)+E_{2}\left(\right|V_{D_{2}}\left|\right)=E_{o}\left(\right|V_{D_{o}}\left|)+\right|V_{c}\left|+\right|V_{D_{o}}\left|-\right|V_{D_{1}}\left|-\right|V_{D_{2}}|,$$

where $|V_{c}|$ denotes the number of common vertices.

In Figure 1, we show the case when the original graph $\Gamma_{o}\left(\right|V_{o}\left|=6,\right|E_{o}\left|=9,\right|V_{D_{o}}\left|=0\right)=\Gamma_{o}\left(6,9,0\right)$ is divided into two subgraphs $\Gamma_{1}\left(4,6,0\right)$ and $\Gamma_{2}\left(4,3,0\right)$. Using Equation (8), one can find that the subgraphs before the disconnection were connected in $|V_{c}|=2$ common vertices. In this relatively simple situation, the generalized Euler characteristics of the graphs or networks can be found from their topological properties, i.e., the numbers of vertices and edges of the graphs. However, if we do not see the graphs and therefore do not know their topological properties but we know their eigenvalues (spectra), the only available solution to the problem is to use Equation (6) to find their generalized Euler characteristics and consequently the number $|V_{c}|$ of the common vertices. The same situation exists for the graphs possessing the Dirichlet boundary conditions. In this case, in order to identify them, one needs to know (measure) the eigenvalues (resonances) of graphs or networks and use Equations (6) and (8) to evaluate the number $|V_{c}|$ of the common vertices.

## 3. Measurements of the Spectra of Microwave Networks

**Figure 2:**
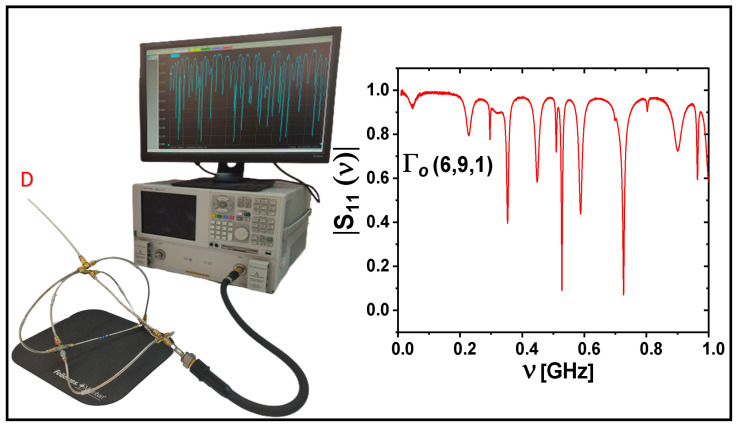
The experimental set-up. It contains an Agilent E8364B vector network analyzer (VNA) and HP 85133-60016 flexible microwave cable that connects the VNA to the measured network. The original microwave network $\Gamma_{o}\left(6,9,1\right)$ possesses a single vertex with the Dirichlet boundary condition, which is marked by the red capital letter *D*. The measured spectrum of the network $\Gamma_{o}\left(6,9,1\right)$ is shown in the inset in the frequency range ν=[0.01,1] GHz.

In order to evaluate the generalized Euler characteristic $E(|V_{D}|)$ defined by Equation (6), we measured the spectra of microwave networks simulating quantum graphs. In our investigations, we used a set-up (see Figure 2) that consisted of an Agilent E8364B vector network analyzer (VNA) and HP 85133-60016 flexible microwave cable that connected the VNA to the measured network. The flexible cable connected to the network is equivalent to attaching an infinite lead to the quantum graph [22,32]. In this way, the one-port scattering matrix $S_{11}\left(\nu \right)$ of the network was measured as a function of microwave frequency ν. The modulus of $|S_{11}\left(\nu \right)|$ was used to identify the network’s resonances. In Figure 2, we also show the original microwave network $\Gamma_{o}\left(6,9,1\right)$, which possesses a single vertex with the Dirichlet boundary condition ($V_{D_{o}}=1$), marked by the red capital letter *D*. The measured spectrum of the network $\Gamma_{o}\left(6,9,1\right)$ is shown in the inset of Figure 2 in the frequency range ν=[0.01,1] GHz. In order to reconfirm our experimental results, the spectra of the quantum graphs simulated by the microwave networks were also calculated numerically using the pseudo-orbits method developed in Ref. [31].

In the construction of microwave networks simulating quantum graphs, we used microwave coaxial cables and junctions that corresponded to the edges and vertices of the quantum graphs. The microwave cables consisted of an outer conductor with an inner radius $r_{2}=0.15$ cm and an inner conductor of radius $r_{1}=0.05$ cm, which was surrounded by the dielectric material (Teflon). The fundamental TEM mode propagates in such cables below the cut-off frequency of the TE${}_{11}$ mode $\nu_{cut}=\frac{c}{\pi \left(r_{1}+r_{2}\right)\sqrt{\varepsilon }}=33$ GHz [70,71], where the dielectric constant of Teflon ε=2.06. It is important to point out that the lengths of edges of the simulated quantum graph have to be compared to the optical lengths of the edges of the microwave networks, i.e., $l_{opt}=\sqrt{\varepsilon }l_{ph}$, where $l_{ph}$ is the physical length of the network edges.

In this paper, we discuss two general situations that are possible when the original network (graph) is split into two subnetworks (subgraphs): the case when the original network and its subnetworks have only the standard boundary conditions and the case when they are characterized by the mixed boundary conditions, when the Dirichlet boundary conditions are present.

### 3.1. Networks with the Standard Boundary Conditions

Here, we will consider the original network $\Gamma_{o}\left(\right|V_{o}\left|,\right|E_{o}\left|,\right|V_{D_{o}}\left|\right)$, which is split into two disconnected subnetworks $\Gamma_{i}\left(\right|V_{i}\left|,\right|E_{i}\left|,\right|V_{D_{i}}\left|\right)$, i=1,2, at the common for the subnetworks vertices $v\in V_{c}$. All networks are characterized by the standard (Neumann) boundary conditions. The experimental realizations of the networks $\Gamma_{o}\left(6,9,0\right)$ and its two subnetworks $\Gamma_{1}\left(4,6,0\right)$ and $\Gamma_{2}\left(4,3,0\right)$ are schematically shown in Figure 1 and Figure 2. In this case, all networks possess only standard (Neumann) boundary conditions, denoted with the capital letter *N*.

The total optical lengths of the networks $\Gamma_{o}\left(6,9,0\right)$, $\Gamma_{1}\left(4,6,0\right)$, and $\Gamma_{2}\left(4,3,0\right)$ are $L_{o}=2.579$ m, $L_{1}=1.675$ m, and $L_{2}=0.940$ m, respectively. The lengths of their shortest edges are $l_{min_{o}}=l_{6}=0.221$ m, $l_{min_{1}}=l_{6}=0.221$ m, and $l_{min_{2}}=l_{9}=0.270$ m, giving $K_{min_{o}}=38$, $K_{min_{1}}=23$, and $K_{min_{2}}=8$, respectively, which were estimated using Equation (7). Experimentally, in order to find the minimum number of resonances determined by the parameters $K_{min_{o}}$, $K_{min_{1}}$, and $K_{min_{2}}$, it was necessary to measure the spectra of the microwave networks $\Gamma_{o}\left(6,9,0\right)$, $\Gamma_{1}\left(4,6,0\right)$, and $\Gamma_{2}\left(4,3,0\right)$ in the frequency ranges [0.010,2.347] GHz, [0.010,2.234] GHz, and [0.010,1.271] GHz, respectively. Taking into account the above parameters, the generalized Euler characteristics $E_{o}\left(\right|V_{D_{o}}\left|\right)$, $E_{1}\left(\right|V_{D_{1}}\left|\right)$, and $E_{2}\left(\right|V_{D_{2}}\left|\right)$ were calculated using Equation (6).

**Figure 3:**
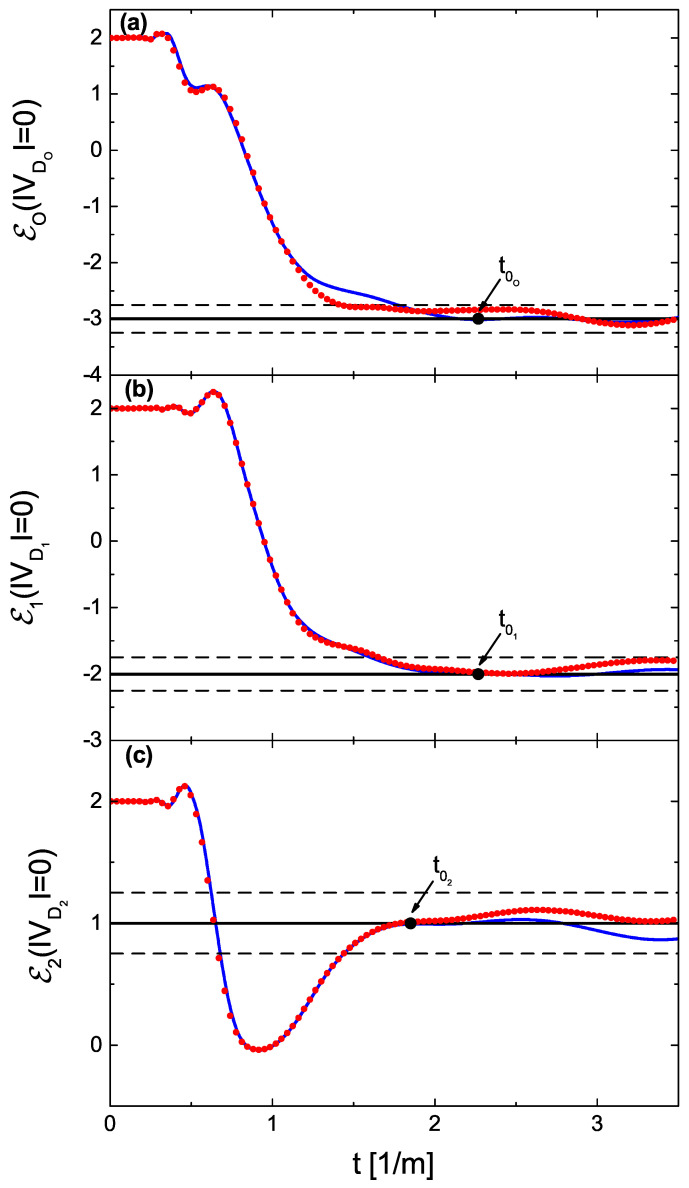
Generalized Euler characteristics evaluated for the networks with the standard boundary conditions as a function of the parameter *t*. Panels (**a**–**c**) show the generalized Euler characteristics $E_{o}\left(\right|V_{D_{o}}\left|=0\right)$, $E_{1}\left(\right|V_{D_{1}}\left|=0\right)$, and $E_{2}\left(\right|V_{D_{2}}\left|=0\right)$ of the networks $\Gamma_{o}\left(6,9,0\right)$, $\Gamma_{1}\left(4,6,0\right)$, and $\Gamma_{2}\left(4,3,0\right)$, respectively. The experimental and numerical results are marked with red dotted and blue full lines, respectively. In all three cases, the plateaus at the generalized Euler characteristics start close to the points $t_{0_{o}}=2.26$ m${}^{-1}$, $t_{0_{1}}=2.26$ m${}^{-1}$, and $t_{0_{2}}=1.85$ m${}^{-1}$, respectively, defined by the theory (see the discussion below Equation (7)). The black broken lines show the limits of the expected errors $E_{q}\left(\right|V_{D_{q}}\left|\right)\pm 1/4$, where q=o,1, and 2.

In Figure 3a–c, we show the generalized Euler characteristics $E_{o}\left(\right|V_{D_{o}}\left|=0\right)$, $E_{1}\left(\right|V_{D_{1}}\left|=0\right)$, and $E_{2}\left(\right|V_{D_{2}}\left|=0\right)$ (red dotted lines), evaluated experimentally as a function of the parameter *t*. The numerically found generalized Euler characteristics are marked with blue full lines. In all three cases, for both experimental and theoretical results, the plateaus at the generalized Euler characteristics start close to the points $t_{0_{o}}=2.26$ m${}^{-1}$, $t_{0_{1}}=2.26$ m${}^{-1}$, and $t_{0_{2}}=1.85$ m${}^{-1}$ defined by the theory (see the discussion below Equation (7)). The values of the generalized Euler characteristics are found to be $E_{o}\left(\right|V_{D_{o}}\left|=0\right)=-3$, $E_{1}\left(\right|V_{D_{1}}\left|=0\right)=-2$, and $E_{2}\left(\right|V_{D_{2}}\left|=0\right)=1$, respectively. Using Equation (8), it is easy to find that $|V_{c}|=2$. It means that, before splitting, the two subgraphs were connected at the two vertices. It is important to point out that the above information was obtained without knowing anything about the topologies of the networks.

### 3.2. Networks with the Mixed Boundary Conditions

We used the same physical networks to investigate the split of the original network $\Gamma_{o}\left(6,9,1\right)$ possessing the mixed boundary conditions into two separated subnetworks $\Gamma_{1}\left(4,6,0\right)$ and $\Gamma_{2}\left(4,3,1\right)$. The network $\Gamma_{o}\left(6,9,1\right)$ and the subnetwork $\Gamma_{2}\left(4,3,1\right)$ possess a single Dirichlet boundary condition. Figure 1 shows the schemes of the networks. The Dirichlet boundary conditions are denoted by the capital letter *D*. All other parameters of the networks, such as the total lengths and the shortest edges, are the same as in the case of the networks with the standard boundary conditions, which were discussed above. However, for the networks with the mixed boundary conditions, one requires the same number of resonances as, in the case of the networks with the Neumann boundary conditions, the frequency ranges where they can be identified are different. For example, for the networks $\Gamma_{o}\left(6,9,1\right)$ and $\Gamma_{2}\left(4,3,1\right)$, they are [0.010,2.500] GHz and [0.010,1.131] GHz, respectively.

**Figure 4:**
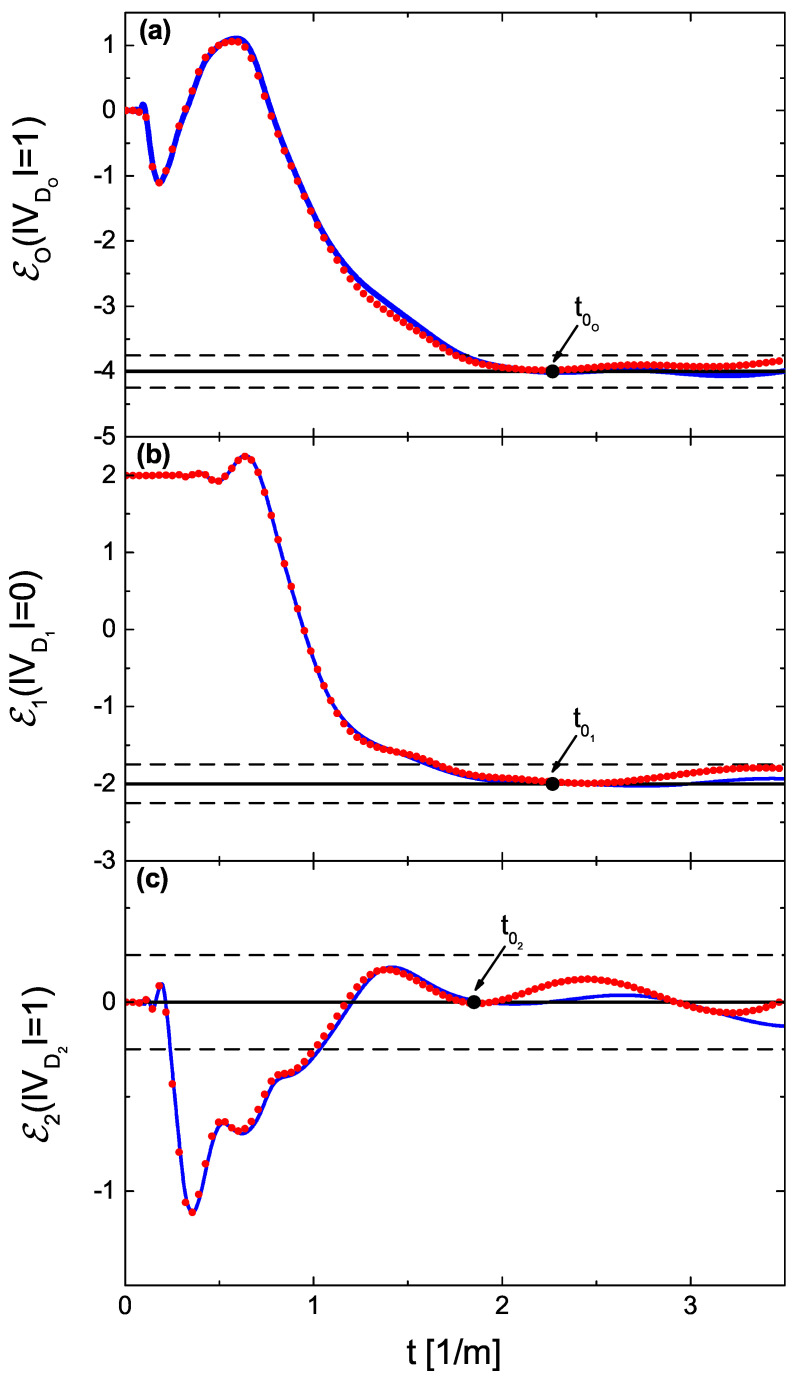
Generalized Euler characteristics evaluated for the networks with the mixed boundary conditions as a function of the parameter *t*. Panels (**a**–**c**) show the generalized Euler characteristics $E_{o}\left(\right|V_{D_{o}}\left|=1\right)$, $E_{1}\left(\right|V_{D_{1}}\left|=0\right)$, and $E_{2}\left(\right|V_{D_{2}}\left|=1\right)$ of the networks $\Gamma_{o}\left(6,9,1\right)$, $\Gamma_{1}\left(4,6,0\right)$, and $\Gamma_{2}\left(4,3,1\right)$, respectively. The experimental and numerical results are marked with red dotted and blue full lines, respectively. Moreover, here, in all three cases, the plateaus at the generalized Euler characteristics start close to the points $t_{0_{o}}=2.26$ m${}^{-1}$, $t_{0_{1}}=2.26$ m${}^{-1}$, and $t_{0_{2}}=1.85$ m${}^{-1}$, respectively, defined by the theory. The black broken lines show the limits of the expected errors $E_{q}\left(\right|V_{D_{q}}\left|\right)\pm 1/4$, where q=o,1, and 2.

In Figure 4a–c, we show the generalized Euler characteristics $E_{o}\left(\right|V_{D_{o}}\left|=1\right)$, $E_{1}\left(\right|V_{D_{1}}\left|=0\right)$, and $E_{2}\left(\right|V_{D_{2}}\left|=1\right)$ (red dotted lines), evaluated experimentally as a function of the parameter *t*. The generalized Euler characteristics that were found numerically are marked with blue full lines. Moreover, here, in all three cases, for both experimental and theoretical results, the plateaus at the generalized Euler characteristics start close to the points $t_{0_{o}}$, $t_{0_{1}}$, and $t_{0_{2}}$ defined by the theory. The values of the generalized Euler characteristics are found to be $E_{o}\left(\right|V_{D_{o}}\left|=1\right)=-4$, $E_{1}\left(\right|V_{D_{1}}\left|=0\right)=-2$, and $E_{2}\left(\right|V_{D_{2}}\left|=1\right)=0$, respectively. In addition, in this case, using Equation (8), we found that $|V_{c}|=2$. One should remark that in the case of the mixed boundary conditions, the knowledge of the topologies of the experimental networks does not allow us to find their generalized Euler characteristics. We also have to know the number of their Dirichlet boundary conditions. Therefore, the measurements of the spectra of the networks and using Equation (6) are mandatory.

## 4. Summary

We analyzed a relationship between the generalized Euler characteristic $E_{o}\left(\right|V_{D_{o}}\left|\right)$ of the original graph (network), which was split into two disconnected subgraphs (subnetworks) i=1,2, and their generalized Euler characteristics $E_{i}\left(\right|V_{D_{i}}\left|\right)$. We showed that the evaluation of the generalized Euler characteristics $E_{o}\left(\right|V_{D_{o}}\left|\right)$ and $E_{i}\left(\right|V_{D_{i}}\left|\right)$ allows us to determine the number $|V_{c}|$ of common vertices where the two subgraphs were initially connected. The theoretical results were numerically verified and confirmed experimentally using microwave networks with the standard and mixed boundary conditions. The application of the generalized Euler characteristics defined by Equation (6) requires the measurement of the spectra of the networks but in return allows us to find $|V_{c}|$ without knowing their topologies. Therefore, it might be possible to apply the properties of the splitting networks discussed in this article in some more practical applications, such as the diagnostics of electronic or microwave networks. One should underline that the first practical test of such diagnostics where the properties of splitting networks and the generalized Euler characteristic were applied was presented in this article. For this purpose, we used real-world systems, such as microwave networks. They are open and dissipative systems, which are completely different from the ideal dissipationless graphs considered in their mathematical studies. In spite of this, even for more complex networks possessing the mixed boundary conditions, we were able to find experimentally the number of common vertices $|V_{c}|$ where the two separated subnetworks were connected before their splitting.

## Data Availability

The data that support the results presented in this paper and other findings of this study are available from the corresponding authors upon reasonable request.
